# Semantic CT features and differentiation model: new primary lung cancer versus metastasis after previous malignancy

**DOI:** 10.1007/s00330-026-12557-w

**Published:** 2026-05-06

**Authors:** Hardeep Singh Kalsi, Kristofer Linton-Reid, Changhyun Kim, Mitchell Chen, Victoria Crowe, Esubalew Alemu, Samir Mahboobani, David Gibeon, Alexander Procter, Mohsen Hajhosseiny, Cara Owens, Emily C. Bartlett, Nuria Porta, Thesha Thavaraja, Simon Doran, Anand Devaraj, Bhupinder Sharma, Arjun Nair, Eric O. Aboagye, Richard W. Lee

**Affiliations:** 1 Early Diagnosis and Detection Centre, Royal Marsden Hospital and Institute for Cancer Research Biomedical Research Centre, London, UK; 2 Department of Surgery and Cancer, Imperial College London, London, UK; 3 Institute for Cancer Research, London, UK; 4 Department of Radiology, Imperial College Healthcare NHS Trust, London, UK; 5 Department of Radiology, Royal Marsden Hospital NHS Foundation Trust, London, UK; 6 Department of Radiology, University College London Hospitals, London, UK; 7 National Heart and Lung Institute, Imperial College London, London, UK; 8 Department of Radiology, Royal Brompton Hospital, London, UK

**Keywords:** CT thorax, Lung lesions after cancer, Second primary lung cancer, Lung metastasis, Semantic radiology feature model

## Abstract

**Objectives:**

New pulmonary lesions after prior cancer present a diagnostic challenge, potentially representing malignancy relapse or new primary lung cancer due to shared risk factors and/or impact of prior oncological therapies. This study evaluated radiologist-defined semantic features for differentiation of second primary lung cancer (SPLC) versus lung metastasis (LM).

**Materials and methods:**

651 single-timepoint, pre-treatment CT thorax scans from the multicentre retrospective AI-SONAR biomarker study (IRAS 331656 REC 23/NE/0151) were divided for review by nine thoracic oncology radiologists to evaluate eight semantic features. Logistic regression analysis was undertaken to identify significant features and a developed ‘Second Malignancy Aetiology Recognition Tool’ model (SMART) was compared to real-world clinical reader performance using McNemar’s test.

**Results:**

649 scans were technically usable, 299 SPLC and 350 LM. Emphysema (*p* < 0.0001, OR 0.20 [95% CI 0.14–0.29]), irregular contour (*p* < 0.0001, OR 0.31 [95% CI 0.20–0.48]) and spiculation (*p* = 0.013, OR 0.51 [95% CI 0.30–0.89]) were more prevalent in SPLC (OR < 1 indicates association with SPLC). Peripheral lung distribution (*p* = 0.003, OR 1.80 [95% CI 1.20–2.68]) was more common in LM (OR > 1 indicates metastasis). SMART model AUC was 0.81 (95% CI 0.78–0.84), LM classification accuracy 75% vs 69% by radiology reader and McNemar *p*-value < 0.01 for comparative accuracy. 550/649 cases were predicted SPLC or LM by radiologists, in which the SMART model LM classification accuracy was 74% vs 77% by reader and McNemar *p*-value 0.20.

**Conclusion:**

In new lesions after prior treated cancer, radiologist readers called SPLC more often than LM. The SMART model performed comparably with expert thoracic radiologists in the diagnosis of LM.

**Key Points:**

***Question*** *Differentiating the malignant aetiology of indeterminate lung lesions after prior cancer presents a growing diagnostic challenge. The literature and nodule guidelines are sparse for this setting.*

***Findings*** *A SMART model derived from semantic CT imaging features correctly classified malignant lung lesions as metastasis or lung cancer, more often than thoracic radiologists.*

***Clinical relevance*** *The SMART model could improve the stratification of malignant new lung lesions after prior cancer. This may lead to earlier diagnosis and optimise patient management, treatment selection and downstream outcomes.*

**Graphical Abstract:**

**Figure:**
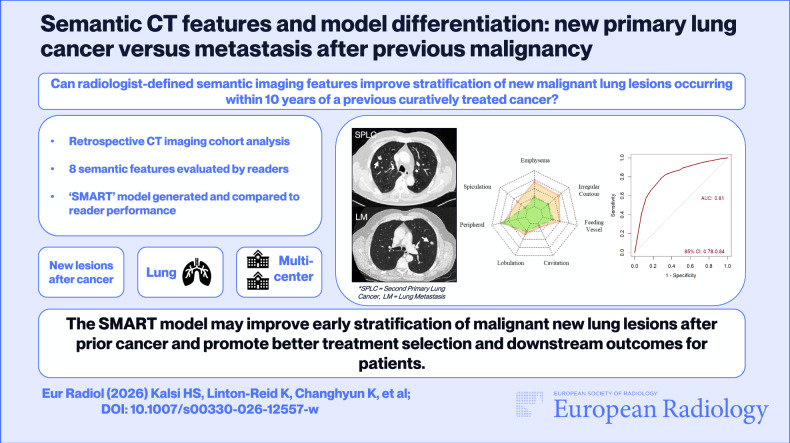

## Introduction

Lung cancer is a leading cause of cancer deaths worldwide, with increasing focus on early diagnosis and implementation of lung cancer screening [1–3]. Increased cross-sectional imaging for post-cancer surveillance and improved patient survival have led to the emergence of a growing cohort of new lung nodules/lesions after prior cancer. Cancer survivors have a higher lifetime incidence of a second cancer episode compared with cancer-naïve cohorts [4, 5]. Therefore, new lung lesions after prior cancer present a diagnostic challenge to radiologists and clinicians, with paucity of data, dedicated guidelines and prediction tools. Current UK lung nodule assessment algorithms (e.g., Brock/Herder) derive from cancer-naïve cohorts [6, 7]. Such models include clinical variables, but imaging features remain core essentials to their predictive outputs. For example, CT imaging features of smoking exposure, e.g., emphysema or airway disease, are expectedly strongly associated with lung cancer [8, 9]. Smoking is also associated with increased risk of extra-thoracic malignancies, e.g., bladder, head and neck [10, 11].

Radiological assessment of pulmonary lesions includes size—a well-established predictor of malignancy—and other morphological characteristics [12–14]. Spiculation, describing spike-like outward peripheral projections into adjacent parenchyma, shows a strong correlation with primary lung malignancy [6, 15]. Lobulation reflects differential growth rates within tumour micro-territories, although the underlying association with cancer compared to benign aetiology is not well validated, with some studies reporting low incidence rates in malignant disease [14, 16]. Lobulated margins, however, were more frequently observed when comparing primary lung cancer versus solitary metastasis in colorectal malignancy [17]. Irregular lesion contour is more commonly observed in primary lung cancer lesions than in solitary metastasis. Conversely, smooth and rounded borders are reported as more common in pulmonary metastasis [17–19].

The significance of vascular connections to pulmonary nodules remains uncertain. A feeding pulmonary artery was more commonly observed in primary lung cancer than in benign lesions [20]. The ‘feeding vessel’ sign has been suggested to relate to metastasis by the provision of a haematogenous dissemination to distal organ sites. Current evidence in the literature, however, remains inconclusive [19–21]. Increased number of pulmonary nodules/lesions frequency may infer increased tendency towards metastasis [22, 23], and additionally when more peripherally located in the lung parenchyma (although no standardised topographical threshold exists) [24]. Cavitation and presence of necrosis are indeterminate, with benign causes such as infection or vasculitis also being attributable [25, 26]. However, squamous cell types of tumours are associated and not solely where the primary origin is lung [27, 28].

Radiology reader variation is an under-recognised problem when evaluating pulmonary lesion assessment. Studies demonstrate inconsistent interpretation, potentially leading to differences in clinical management [29–31]. In patients with a prior history of cancer, management of lung metastasis versus new lung cancer differs considerably. Lung nodule classification therefore affects treatment selection and patient outcome. Clinical uncertainty may also increase healthcare resource use due to additional scans and exacerbate patient anxiety.

Characterisation of morphological characteristics and their role in discriminating between new lung cancer or pulmonary metastasis following prior radically treated cancer is poorly described [32]. We evaluate radiologist-defined semantic CT imaging features to assess the hypothesis that structured radiology reporting improves the stratification of malignant lesions.

## Materials and methods

### Data curation

**Fig. 1:**
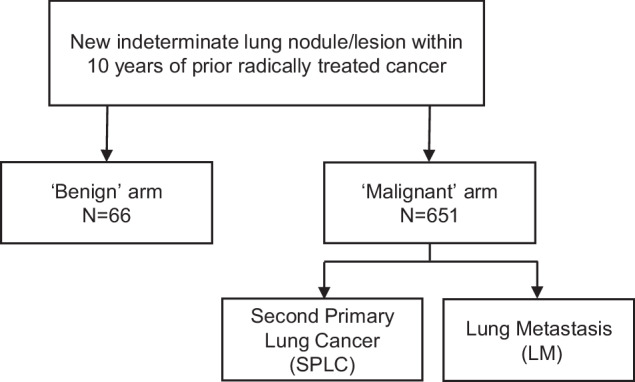
AI-SONAR study (ClinicalTrials.gov ID NCT05375591) dataset overview of recruitment arms (*N* = 651). Ground truth confirmed by either biopsy or multidisciplinary team consensus ± sequential CT/nuclear medicine imaging (see Supplementary Material)

Single timepoint thoracic CT imaging scans of 651 cases of new lung lesions presenting within 10 years of a previously radically treated cancer were curated within the ‘Malignant’ arm of the AI & radiomics for Stratification Of lung Nodules After Radically treated cancer (AI-SONAR) retrospective imaging biomarker study (ClinicalTrials.gov ID NCT05375591) shown in Fig. 1. Cases identified were split into 2 classes (1) Second primary lung cancer (SPLC), (2) Lung metastasis (LM). Curated scans were 2.5 mm slice thickness or less, and either (1) first index scan demonstrating detection of a new lung lesion or (2) first available imaging scan on records of a visible new lung lesion. All curated scans were undertaken prior to any subsequent targeted therapy. Ground truth was determined either via positive biopsy or multidisciplinary team consensus, including interval CT assessment ± nuclear medicine imaging (see Supplementary Material). The region of interest (ROI) was determined as a lesion that, in real-world clinical practice, was responsible for diagnosis either through biopsy sampling or MDT consensus review. This identification process was confirmed following an extensive review of electronic healthcare record data and sequential imaging.

### Ethics

Health Regulatory Authority (HRA) approval (IRAS 331656) was given with Research Ethics Committee (REC) favourable opinion (23/NE/015)1 for the AI-SONAR study. Imaging scans were collected as part of routine clinical care, and patient consent was not required.

### Imaging data processing and handling

Following case identification and recruitment, imaging scans were anonymised locally to remove patient identifiers and uploaded from NHS institutions to the Xtensible Neuroimaging Assessment Tool (XNAT) platform in DICOM format (Digital Imaging and Communications in Medicine). Imaging scans were downloaded and converted to NIFTI format (Neuroimaging Informatics Technology Initiative) using a ‘dcm2niix’ (https://github.com/rordenlab/dcm2niix) in Jupyter Python Notebook version 3.9.2. Imaging scans were resampled to 1 × 1 × 1 mm voxel parameter for analysis, in line with the median/mean slice thickness across the dataset. All scan ‘study IDs’ were randomised and given a new anonymous ‘Reading ID’ (1–651) to avoid reader recall bias. ROI was highlighted using a red label marker in the application ITK-SNAP^TM^, with pulmonary lobe location denoted (e.g., RLL_ROI). All ROIs were identified as a lesion which was assessed in real-world clinical practice to determine a diagnosis of SPLC or LM, irrespective of other imaging findings and whether the ROI was sampled or not.

### Radiology reading

**Fig. 2:**
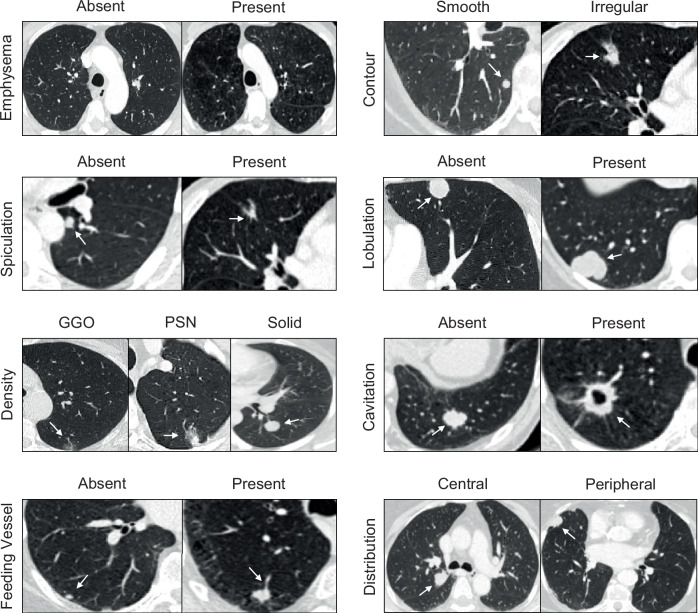
Radiologist-defined semantic features assessed on CT imaging from top to bottom row with arrow highlighting ROI: emphysema (absent/present), contour type (smooth/irregular), spiculation (absent/present), lobulation (absent/present), density (GGO/PSN/solid), cavitation (absent/present), feeding vessel (absent/present), distribution* (central/peripheral). GGO, ground glass opacity; PSN, part-solid nodule; ROI, region of interest

Following literature review and expert thoracic oncology radiology panel consensus (A.D., A.N., B.S.), eight semantic CT features were identified for assessment: presence of emphysema, lesion contour, spiculation, lobulation, density, cavitation, feeding vessel and distribution in addition to a radiologist ‘clinical classification as shown in Fig. 2. Central vs peripheral ROI distribution was determined using a proximal bronchial tree threshold outlined by the UK Stereotactic Ablative Radiotherapy Consortium (SABR) [33]. Nine radiologist readers (V.C., M.C., E.A., S.M., A.P., D.G., M.H., C.O., E.B.) were recruited to carry out reading of the dataset and reporting on the selected features. All readers were UK FRCR-accredited, comprising 2 specialist training registrars (average experience 5 years), 2 post-CCT fellows (average experience 7.5 years) and 5 consultants (average experience 10 years). All readers worked in tertiary thoracic oncology centres.

**Fig. 3:**
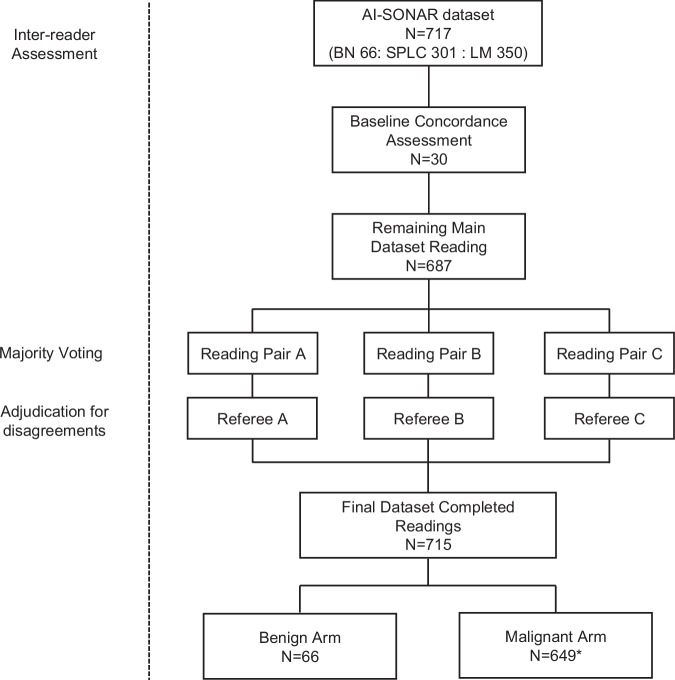
Radiologist reporting of semantic features on AI-SONAR dataset (ClinicalTrials.org NCT05375591), *N* = 717. BN, benign; SPLC, second primary lung cancer; LM, lung metastasis. * Two cases from original *N* = 651 excluded as not technically usable

Six readers were grouped into pairs, with the remaining three readers each acting as an adjudicator for reading disagreements for one reading pair (Fig. 3). Radiologists were required to view and interpret scans using the lung window setting (width, 1500 HU: level, −600 HU) in ITK-SNAP^TM^. Semantic features were assessed on ROI alone; however, the entire thoracic field anatomy was reviewed for ‘clinical classification’. Majority voting was used to determine final reported outcomes per semantic feature per case amongst reading pairs.

A baseline concordance assessment of semantic features was conducted for all readers on a selected set of 30 cases, which comprised 8 positive benign (BN) control cases (derived from the ‘Benign’ cohort of the AI-SONAR study), 11 SPLC and 11 LM. This sample number was determined following consultation with a statistician (NP) with experience in imaging-based research and clinical reader interpretation studies. Individual reader vs reader (1–9) agreement analysis was additionally evaluated (see Supplementary Material).

### Statistical analysis

R version 4.4.2 (R Project for Statistical Computing, http://www.r-project.org) was used for all statistical analyses. Fleiss Kappa calculation was performed for each semantic feature to assess for multiple inter-reader agreement in the baseline concordance assessment. Cohen’s kappa assessment was carried out for subsequent individual reading pair agreement analysis in the main dataset.

After completion of all dataset assessment and referee adjudication, categorical outputs were converted into binary ‘0 or 1’ for each feature and ‘0, 1 or 2’ for outcomes relating to lesion density. *T*-test analysis was performed for continuous data variables between groups and Chi-squared test for categorical variables. Univariate and multivariate logistic regression (LR) was performed using the ‘log’ package to determine (1) significant semantic features relevant to lesional class, (2) significant semantic features relevant to lesional class when balanced against all other features. Adjusted *p*-values were determined using Benjamini–Hochberg correction to reduce the false discovery rate.

A multivariate LR model based on semantic features of significance, named ‘Second Malignancy Aetiology Recognition Tool’ (SMART), was generated using the ‘PASWR’ and ‘rms’ packages. Area under the curve (AUC) performance was assessed using the ‘pROC’ package, and model performance was evaluated against reader clinical classification (SPLC vs LM) using a confusion matrix approach. McNemar *p*-value was calculated to compare the accuracy of the SMART model versus the reader’s clinical diagnosis on the dataset.

## Results

### Dataset demographics

651 cases and corresponding CT scans were identified, with 649 technically usable and two cases of SPLC excluded due to an inability to reliably visualise an ROI and perform accurate reading. 299 cases were SPLCs, and 350 were LMs. CT scans were acquired from two host institutions (RMH, RBH) with patient and imaging demographics of all cases shown in Table 1.

**Table 1:** Patient and imaging demographics of malignant cohort from AI-SONAR study (ClinicalTrials.org NCT05375591), *N* = 649

Demographic		SPLC *N* = 299	LM *N* = 350	*p*-value
Age (years)	Mean (SD)	72.3 (9.1)	63.3 (13.2)	< 0.0001
	Interquartile range	10.2	18.5	
Gender (*N*, %)	Male	159 (53.2%)	166 (47.4%)	0.17
	Female	140 (46.8%)	184 (52.6%)	
Previous FPC type (*N*, %)	Bladder	20 (6.7%)	7 (2.0%)	< 0.0001
	Breast	55 (18.4%)	25 (7.1%)	
	Colorectal	21 (7.0%)	55 (15.7%)	
	Gastrointestinal stromal tumour	1 (0.3%)	0 (0.0%)	
	Gynaecological	7 (2.3%)	56 (16%)	
	Head and neck	34 (11.4%)	38 (10.9%)	
	Hepatobiliary/upper GI	6 (2.0%)	13 (3.7%)	
	Lung and mesothelioma	74 (24.7%)	42 (12.0%)	
	Lymphoma	5 (1.7%)	3 (0.9%)	
	Prostate	41 (13.7%)	7 (2.0%)	
	Renal	5 (1.7%)	27 (7.7%)	
	Sarcoma	5 (1.7%)	42 (12.0%)	
	Skin	22 (7.4%)	29 (8.3%)	
	Testicular	0 (0.0%)	3 (0.9%)	
	Thymic/thymoma	1 (0.3%)	1 (0.3%)	
	Thyroid	2 (0.7%)	2 (0.6%)	
FPC nodal status (*N*, %)	Positive	78 (26.1%)	113 (32.3%)	0.09
	Negative	221 (73.9%)	237 (67.7%)	
Lesion size (mm)	Mean (SD)	34.7 (25.7)	18.9 (17.8)	< 0.0001
	Interquartile range	34.5	15.0	
Lesion frequency (*N*, %)	Single	212 (70.9%)	159 (45.4%)	< 0.0001
	Multiple	77 (29.1%)	191 (54.6%)	
Time after FPC (days)	Mean (SD)	1597.1 (974.4)	990.0 (795.0)	< 0.0001
	Interquartile range	1521.1	945.3	
Route to detection (*N*, %)	Asymptomatic screening	3 (1.0%)	0 (0.0%)	< 0.0001
	Incidental finding	33 (11.0%)	9 (2.6%)	
	New symptom onset	148 (49.5%)	92 (26.3%)	
	Post-cancer imaging surveillance	115 (38.5%)	249 (71.1%)	
Biopsy diagnosis (*N*, %)	Yes	259 (86.6%)	216 (61.7%)	< 0.0001
	No (MDT consensus)	40 (13.4%)	134 (38.3%)	
CT slice thickness (mm)	Mean (median)	1.2 (1.0)	1.1 (1.0)	
Contrast present (*N*, %)	Yes	234 (78.2%)	296 (84.6%)	0.05
	No	65 (21.8%)	54 (15.4%)	
Imaging source (*N*, %)	Native institution	82 (27.4%)	171 (48.9%)	< 0.0001
	External import	217 (72.6%)	179 (51.1%)	

*CT* computer tomography, *FPC* first primary cancer, *LM* lung metastasis, *MDT* multidisciplinary team, *SD* standard deviation, *SPLC* second primary lung cancer

### Reader agreement

Baseline reader concordance assessment with Fleiss Kappa analysis for multi-reader agreement is shown in Table 2. This demonstrated ‘Fair’ agreement for all readers for the interpretation of emphysema, lobulation, density and feeding vessel. ‘Moderate’ agreement was observed in reader interpretation of contour, spiculation, distribution and clinical classification. ‘Almost perfect’ agreement was measured for the interpretation of cavitation. Cohen’s kappa inter-reader agreement analysis for each ‘reader vs reader’ per semantic feature in the baseline concordance assessment and main dataset reading by subsequent individual reading pairs is outlined in Supplementary Material.

**Table 2:** Baseline reader concordance assessment (**a**) and interpretation of Fleiss Kappa values for multiple inter-reader agreement levels (**b**)

a. Baseline reader concordance assessment		
Semantic radiology feature	Fl-K (95% CI)	Agreement level
Emphysema	0.38 (0.32–0.44)	Fair
Contour	0.42 (0.36–0.48)	Moderate
Spiculation	0.51 (0.45–0.57)	Moderate
Lobulation	0.27 (0.22–0.33)	Fair
Density	0.39 (0.33–0.44)	Fair
Cavitation	0.89 (0.83–0.94)	Almost perfect
Feeding vessel	0.22 (0.16–0.28)	Fair
Distribution	0.58 (0.52–0.64)	Moderate
Clinical classification	0.44 (0.39–0.48)	Moderate

b. Interpretation of Fleiss Kappa values for multiple inter-reader agreement levels	
Kappa value	Interpretation
1	Perfect
0.81–0.99	Almost perfect
0.61–0.8	Substantial
0.41–0.6	Moderate
0.21–0.4	Fair
0.1–0.2	Slight
0	None

Fleiss Kappa scores for multiple inter-reader agreement assessments on the baseline reading set, *N* = 30

*FL-K* Fleiss Kappa, *CI* confidence interval

### Semantic features of SPLC and LM

Comparison between SPLC and LM for all semantic features is shown in Table 3. Univariate LR followed by Benjamini–Hochberg *p*-value correction demonstrated all semantic features to show significance in differentiating between SPLC and LM (Table 4). Multivariate LR followed by Benjamini–Hochberg *p*-value correction demonstrated emphysema (*p*-value < 0.001), contour (*p*-value < 0.0001), distribution (*p*-value < 0.01) and spiculation (*p*-value = 0.013) to be statistically significant in discriminating between SPLC and LM aetiology (Table 4).

**Table 3:** Distribution of reported semantic features per each outcome class in the malignant cohort, *N* = 649

Semantic feature	Outcome	SPLC (*N* = 299)		LM (*N* = 350)	
		No	% Total	No	% Total
Emphysema	Yes	206	69.2%	93	26.6%
	No	93	30.8%	257	73.4%
Contour	Smooth	102	34.1%	262	74.9%
	Irregular	197	65.9%	88	25.1%
Spiculation	Yes	106	35.5%	33	9.4%
	No	193	64.5%	317	90.6%
Lobulation	Yes	103	34.4%	91	26.0%
	No	196	65.6%	259	74.0%
Density	GGO	12	4.0%	1	0.3%
	PSN	15	5.0%	7	2.0%
	Solid	272	91.0%	342	97.7%
Cavitation	Yes	37	12.4%	20	5.7%
	No	262	87.6%	330	94.3%
Feeding vessel	Yes	149	49.8%	107	30.6%
	No	150	50.2%	243	69.4%
Distribution	Central	107	35.8%	95	27.1%
	Peripheral	192	64.2%	255	72.9%

*GGO* ground glass opacity, *LM* lung metastasis, *PSN* part-solid nodule, *ROI* region of interest, *SPLC* second primary lung cancer

**Table 4:** Logistic regression (LR) for each semantic feature in relation to outcome class in malignant cohort (0 = SPLC, 1 = LM), *N* = 649 (299 SPLC:350 LM)

Semantic feature	Univariate LR				Multivariate LR			
	LR Est	OR (95% CI)	*p*-value	Adj-*p*	LR Est	OR (95% CI)	*p*-value	Adj-*p*
Emphysema	−1.81	0.16 (0.12–0.23)	< 0.0001	< 0.0001	−1.61	0.20 (0.14–0.29)	< 0.0001	< 0.0001
Contour	−1.75	0.17 (0.12–0.24)	< 0.0001	< 0.0001	−1.18	0.31 (0.20–0.48)	< 0.0001	< 0.0001
Spiculation	−1.66	0.19 (0.12–0.29)	< 0.0001	< 0.0001	−0.67	0.51 (0.30–0.89)	0.013	0.013
Lobulation	−0.04	0.69 (0.47–0.94)	< 0.05	< 0.05	−0.31	0.73 (0.49–1.09)	0.13	0.18
Cavitation	−0.85	0.43 (0.24–0.76)	< 0.05	< 0.01	−0.32	0.73 (0.36–1.45)	0.36	0.42
Feeding vessel	−0.08	0.44 (0.32–0.61)	< 0.0001	< 0.0001	−0.08	0.93 (0.62–1.38)	0.71	0.71
Distribution	+0.40	1.50 (1.07–2.09)	< 0.05	< 0.05	+0.59	1.80 (1.2–2.68)	0.003	0.003

Adjusted *p*-value correction using the Benjamini–Hochberg method

*Adj-p* adjusted *p*-value, *CI* confidence interval, *LM* lung metastasis, *LR Est* logistic regression estimate, *OR* odds ratio, *ROI* region of interest, *SPLC* second primary lung cancer

### SMART model and clinician benchmarking

**Fig. 4:**
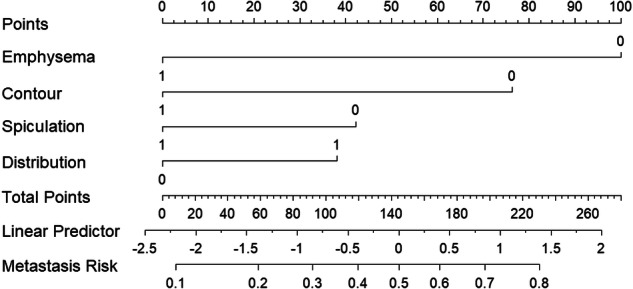
SMART (Second Malignancy Assessment Recognition Tool) nomogram model for classification of lung metastasis (LM) vs second primary lung cancer (SPLC). Selected semantic features of significance following multivariate logistic regression analysis. Key: emphysema (absent = 0, present = 1), contour (smooth = 0, irregular = 1), spiculation (absent = 0, present = 1), distribution (central = 0, peripheral = 1)

**Fig. 5:**
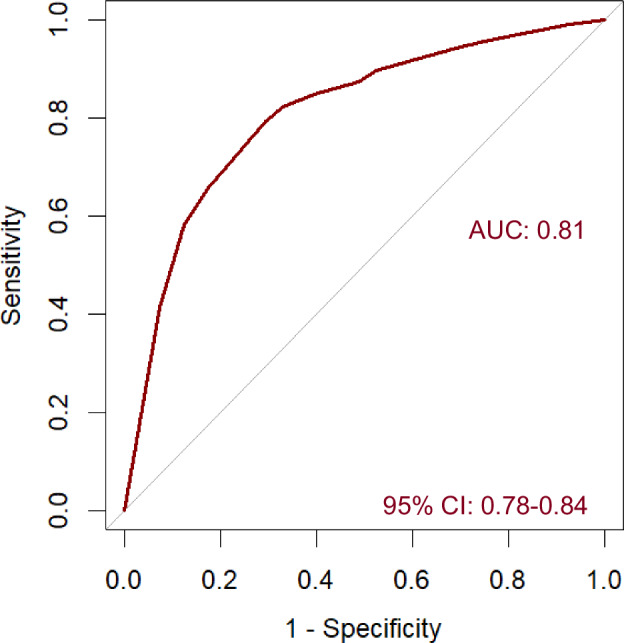
Receiver operating characteristic (ROC) curve for the SMART model based on the semantic features of significance. AUC, area under the curve; CI, confidence interval

**Fig. 6:**
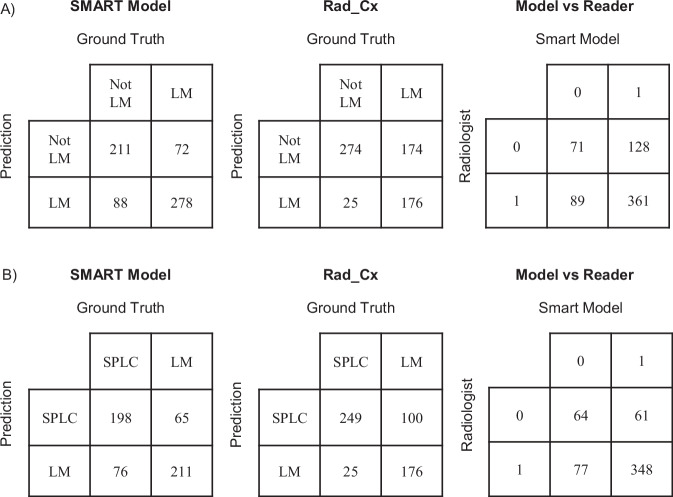
Confusion matrices for SMART model vs clinical reader classification (Rad_Cx) and comparison of relative diagnostic accuracy (0 = SPLC, 1 = LM). **A** Classification of LM in the malignant cohort (*N* = 649), **B** classification of SPLC vs LM where SPLC or LM was diagnosed by the reader in the malignant cohort (*N* = 550). LM, lung metastasis; ROI, region of interest; SPLC, second primary lung cancer

Using semantic features selected following multivariate LR, a nomogram model based on presence of emphysema, contour type, presence of spiculation and distribution type was generated termed ‘Second Malignancy Aetiology Recognition Tool’ or ‘SMART’ as demonstrated in Fig. 4. The SMART model achieved an AUC of 0.81 (95% CI 0.78–0.84), sensitivity 79.4%, specificity 70.6%, diagnostic accuracy 75% and F1 score of 0.78 as shown in Fig. 5. Model performance versus radiology reader is shown in Table 5 with confusion matrix breakdown and diagnostic accuracy comparison in Fig. 6. Comparing the SMART model to readers in lung metastasis classification (1 = LM, 0 = Not LM), the model provided a classification accuracy of 75% vs 69%, sensitivity 79% vs 50%, specificity 71% vs 92% and F1 score of model precision recall of 0.78 vs 0.64. 49.7% (174/350) of LM cases were misclassified as ‘not metastasis’ by radiologists versus 20.6% mislabelled (72/350) by the SMART model. McNemar *p*-value was < 0.01 when comparing the classification accuracy of the SMART model versus radiology readers. In 71/649 cases, both the SMART model and reader classification of LM were incorrect versus 361/649 cases where both were correct. The SMART model was incorrect in 89/649 cases where reader’s interpretation was accurate. Conversely, in 128/649 cases, classification by radiology readers was incorrect where SMART model classification was accurate.

**Table 5:** Comparing the classification performance of the SMART model versus clinical reader classification (Rad_Cx) of lung metastasis (LM = 1, not LM = 0) in the malignant cohort (*N* = 649), and where SPLC or LM was diagnosed by a radiologist in the malignant cohort (*N* = 550)

Performance metric	All ROIs (*N* = 649)		‘SPLC or LM’ diagnosis by reader (*N* = 550)	
	SMART	Rad_Cx	SMART	Rad_Cx
Accuracy	0.754	0.693	0.744	0.773
Sensitivity	0.794	0.503	0.765	0.638
Specificity	0.706	0.916	0.723	0.909
PPV	0.760	0.876	0.735	0.876
NPV	0.746	0.612	0.753	0.714
F1 score	0.780	0.640	0.750	0.738

*LM* lung metastasis, *NPV* negative predictive value, *PPV* positive predictive value, *ROI* region of interest, *SPLC* second primary lung cancer

As part of the wider AI-SONAR study radiology reading exercise, radiologist reads were collated on benign cases, SPLC and LM cases. Therefore, 550/649 cases were labelled as either SPLC or LM by radiologists and 99 as benign. In the analysis of this sub-group where SPLC or LM was labelled by readers, the SMART model achieved a classification accuracy of 74% versus 77% (0 = SPLC, 1 = LM), sensitivity 77% vs 64%, specificity 72% vs 91% and F1 score 0.75 vs 0.74 as shown in Table 5, with confusion matrix breakdown in Fig. 6. 36.2% (100/276) of LM cases were mislabelled as SPLC by radiology readers versus 23.6% (65/276) by the SMART model. 72.3% (198/274) of SPLC were correctly classified by the SMART model in comparison to 90.8% (249/274) by the radiologist reader. McNemar *p*-value was 0.20 when comparing the classification accuracy of the SMART versus the radiology reader. In 64/550 cases, both the SMART model and radiology readers were wrong versus 348/550 cases where both were correct. In 61/550 cases, reader interpretation was incorrect but SMART model classification was correct, compared to 77/550 cases where the SMART model was incorrect but reader interpretation was accurate.

## Discussion

Determining the aetiology of new lung lesions in patients with a prior history of radically treated cancer remains a diagnostic challenge with paucity of evidence and clinical guidelines in the literature specific to this group, where the rate of metastasis influences the decision-making and outcome. This particular cohort of patients is expected to grow due to improved cancer survival. The importance of early, accurate diagnosis is vital to ensure prompt and correct management and promote the best outcomes for individuals.

To the authors’ knowledge, large studies evaluating semantic features have not previously been performed on a dataset of second primary lung cancer and metastatic lung lesions occurring after prior radically treated cancer. This is the first reported study where a semantic feature model (SMART) has been benchmarked directly against thoracic radiologist classification (second primary lung cancer versus metastasis) and demonstrated comparable performance. Importantly, all assessments were made on single-timepoint thoracic CT scans. Radiologist and model performance may differ with the availability of temporal scan data. Further evaluation may benefit from an implementation of a ‘confidence level’ statement when predicting underlying lesion aetiology or evaluation of sequential imaging scans.

Radiologist readers generally consistently undercalled metastatic lung lesions, potentially suggesting an increased need for data to underpin decision-making, and an opportunity for decision support tools such as the SMART model and those based on radiomics and AI. A potential hypothesis for this predilection is a default position of SPLC-bias when diagnostic certainty is unclear and risk-averse behaviour by readers. Furthermore, this may reflect a culture of ‘benefit of doubt’ decision-making based on perceived treatment options. Further research is required to substantiate this, since there are implications for potential over-investigation, including radiation exposure, increased imaging resource burden, biopsy risk and false survival projections. We recognise that instructing readers to systematically assess specific semantic features may have influenced diagnostic classification.

The class balance reflects the real-world nature and strict recruitment criteria in the AI-SONAR study (see Supplementary Material). Reasons for cases not being included were due to the following reasons: (1) CT slice thickness sub-optimal, (2) pre-treatment target scan of interest not available, (3) technical difficulties identifying ROI due to adjacent structures (e.g., lobar collapse) or lack of contrast enhancement. The heterogeneity of the scans curated in two tertiary centres but imported from multiple referral hospitals presents a strength of the dataset and improves the generalisability of the data. Whilst it is recognised that kernel reconstructions may differ depending on scanner acquisition protocols or vendors, voxel dimensions and readings were standardised.

Variation in radiologist assessment is recognised. When we assessed how semantic feature differentiation might account for inter-reader variability, we observed that some features are more consistently interpreted across readers than others. Lesion size, absence of contrast administration, lesion location or proximity to adjacent structures may influence ease of reading. For example, ‘presence’ of a feeding vessel requires assessment of whether it directly leads into a lesion or is by chance merely regionally associated due to a lesion’s size or anatomical topography.

This presents the real-world deployment opportunity for our model. Standardisation of clinical management, to avoid misclassification of primary lung cancer versus metastasis, and potentially selection of curative treatment versus accurate systemic treatment presents an opportunity for improved patient care and outcomes. We recognise that the subjectivity of certain features presents important implications for deployment. The AI-SONAR study adhered to strict recruitment criteria, but further work is needed to understand how reader variability impacts model performance in a wider setting, noting that a standardised model such as ‘SMART’ may also mitigate for real-world variation in reader behaviour and lead to improved consistency of decision-making.

A further potential limitation is the decision to not exclude lesions below/above a specific size to maximise cases available and enrich the data with real-world heterogeneity. Further work may benefit from the analysis of defined size criteria-based populations. 94.6% of lesions were solid type density, therefore limiting analysis. Where the dataset composition includes more ground glass or part-solid lesions, further evaluation would be pertinent.

All readers worked in tertiary thoracic oncology centres, and whilst the authors recognise concerns about low reproducibility of semantic features and heterogeneity in assessment, the SMART model takes this into account and presents a standardised assessment tool independent of human reader variability. Composite variables can offer a stronger signal and greater predictive power than individual variables, as they combine multiple data points into a single, more comprehensive metric that can capture underlying constructs and complex relationships more effectively, reducing noise and increasing the overall information content for analytical models.

In this work, we chose to retain focus on imaging features, and so clinical factors were excluded, since we anticipated that imaging traits were likely to sufficiently represent other clinic-demographic features and retain the simplicity of a scan-only algorithm. The mean age was observed to be higher in the SPLC group compared to the LM cohort. Certain cancer types are more common in specific age groups, with lung cancer being more common in older age [34]. Whilst we do not have sufficient data to rule out age as a confounding factor in this novel patient cohort, further research is indicated into the impact of age on the likelihood of a given cancer type in this cohort (SPLC vs LM), and whether including clinical variables can improve model performance.

The SMART model was developed on the AI-SONAR dataset and evaluated by clinicians using the same data. The authors recognise that whilst performance outputs are encouraging, a more accurate validation of the model would be achieved using an independent external test set. Additionally, site-based split analysis was not possible as both recruiting institutions (RMH, RBH) received referrals from a wide range of secondary care centres, and as such, individual sample numbers were too low to use as a holdout validation dataset. Bootstrapping or cross validation were not undertaken due to a lack of feasibility, but the authors recognise this as an avenue for future analysis.

Significant study has been undertaken evaluating radiomics-based models for pulmonary malignancy prediction with some promising success. Such deep learning models validate this approach for effective malignancy prediction in retrospective cohorts, but do not address the specific problem of lung nodule radiomics after prior malignancy [35, 36].

In conclusion, this study presents important data on the semantic CT imaging features of SPLC vs LM and postulates that a structured approach to imaging interpretation of new lung nodules after prior radically treated cancer may assist and improve diagnostic classification of second cancers of the lung versus metastasis. Further work may involve exploring a deep learning-based model using automated interpretation of semantic features and evaluating predictive classification outcomes versus (1) clinical readers and (2) SMART model predictions. Development of an automated pipeline might help expedite earlier clinical decision-making, optimise use of imaging resources and improve prognostic outcomes. Additional exploratory work of interest would involve integration of clinical variables based on patient history (e.g., smoking status, previous cancer type) and use of radiomic features with machine learning approaches for the development of a multimodal classification model.

## Supplementary information

Supplementary Material

## Abbreviations

AI-SONAR: Artificial Intelligence & radiomics for Stratification Of lung Nodules After Radically treated cancer
AUC: Area under the curve
CI: Confidence interval
LM: Lung metastasis
LR: Logistic regression
RBH: Royal Brompton Hospital, United Kingdom
RMH: Royal Marsden Hospital NHS Foundation Trust, United Kingdom
ROI: Region of interest
SMART: Second malignancy assessment recognition tool
SPLC: Second primary lung cancer

## References

[1] Sung H, Ferlay J, Siegel RL et al (2021) Global cancer statistics 2020: GLOBOCAN estimates of incidence and mortality worldwide for 36 cancers in 185 countries. CA Cancer J Clin 71:209–249. 10.3322/caac.21660 33538338 10.3322/caac.21660

[2] Thandra KC, Barsouk A, Saginala K, Aluru JS, Barsouk A (2021) Epidemiology of lung cancer. Contemp Oncol 25:45–52. 10.5114/wo.2021.103829 10.5114/wo.2021.103829 PMC8063897 33911981

[3] O’Dowd EL, Lee RW, Akram AR et al (2023) Defining the road map to a UK national lung cancer screening programme. Lancet Oncol 24:e207–e218. 10.1016/S1470-2045(23)00104-3 37142382 10.1016/S1470-2045(23)00104-3

[4] Ruan X, Huang D, Zhan Y et al (2023) Risk of second primary cancers after a diagnosis of first primary cancer: a pan-cancer analysis and Mendelian randomization study. Elife 12:e86379. 10.7554/eLife.86379 37917154 10.7554/eLife.86379 PMC10622143

[5] Barclay ME, Lyratzopoulos G, Walter FM, Jefferies S, Peake MD, Rintoul RC (2019) Incidence of second and higher order smoking-related primary cancers following lung cancer: a population-based cohort study. Thorax 74:466–472. 10.1136/thoraxjnl-2018-212456 30777897 10.1136/thoraxjnl-2018-212456 PMC6475108

[6] McWilliams A, Tammemagi MC, Mayo JR et al (2013) Probability of cancer in pulmonary nodules detected on first screening CT. N Engl J Med 369:910–919. 10.1056/NEJMoa1214726 24004118 10.1056/NEJMoa1214726 PMC3951177

[7] Herder GJ, van Tinteren H, Golding RP et al (2005) Clinical prediction model to characterize pulmonary nodules: validation and added value of 18F-fluorodeoxyglucose positron emission tomography. Chest 128:2490–2496. 10.1378/chest.128.4.2490 16236914 10.1378/chest.128.4.2490

[8] Zhang P, Chen PL, Li ZH et al (2022) Association of smoking and polygenic risk with the incidence of lung cancer: a prospective cohort study. Br J Cancer 126:1637–1646. 10.1038/s41416-022-01736-3 35194190 10.1038/s41416-022-01736-3 PMC9130319

[9] Li Y, Swensen SJ, Karabekmez LG et al (2011) Effect of emphysema on lung cancer risk in smokers: a computed tomography-based assessment. Cancer Prev Res 4:43–50. 10.1158/1940-6207.CAPR-10-0151 10.1158/1940-6207.CAPR-10-0151 PMC3018159 21119049

[10] Weber MF, Sarich PEA, Vaneckova P et al (2021) Cancer incidence and cancer death in relation to tobacco smoking in a population-based Australian cohort study. Int J Cancer 149:1076–1088. 10.1002/ijc.33685 34015143 10.1002/ijc.33685

[11] Kulhánová I, Forman D, Vignat J et al (2020) Tobacco-related cancers in Europe: the scale of the epidemic in 2018. Eur J Cancer 139:27–36. 10.1016/j.ejca.2020.07.024 32957011 10.1016/j.ejca.2020.07.024

[12] Callister ME, Baldwin DR, Akram AR et al (2015) British Thoracic Society guidelines for the investigation and management of pulmonary nodules. Thorax 70:ii1–ii54. 10.1136/thoraxjnl-2015-207168 26082159 10.1136/thoraxjnl-2015-207168

[13] Devaraj A, van Ginneken B, Nair A, Baldwin D (2017) Use of volumetry for lung nodule management: theory and practice. Radiology 284:630–644. 10.1148/radiol.2017151022 28825886 10.1148/radiol.2017151022

[14] Gould MK, Donington J, Lynch WR et al (2013) Evaluation of individuals with pulmonary nodules: when is it lung cancer? Diagnosis and management of lung cancer, 3rd ed: American College of Chest Physicians evidence-based clinical practice guidelines. Chest 143:e93S–e120S. 10.1378/chest.12-2351 23649456 10.1378/chest.12-2351 PMC3749714

[15] Winer-Muram HT (2006) The solitary pulmonary nodule. Radiology 239:34–49. 10.1148/radiol.2391050343 16567482 10.1148/radiol.2391050343

[16] Snoeckx A, Reyntiens P, Desbuquoit D et al (2018) Evaluation of the solitary pulmonary nodule: size matters, but do not ignore the power of morphology. Insights Imaging 9:73–86. 10.1007/s13244-017-0581-2 29143191 10.1007/s13244-017-0581-2 PMC5825309

[17] Lee JE, Jeong WG, Kim YH (2021) Differentiation of primary lung cancer from solitary lung metastasis in patients with colorectal cancer: a retrospective cohort study. World J Surg Oncol 19:28. 10.1186/s12957-021-02131-7 33487164 10.1186/s12957-021-02131-7 PMC7831192

[18] Seo JB, Im JG, Goo JM, Chung MJ, Kim MY (2001) Atypical pulmonary metastases: spectrum of radiologic findings. Radiographics 21:403–417. 10.1148/radiographics.21.2.g01mr17403 11259704 10.1148/radiographics.21.2.g01mr17403

[19] Stadelmann SA, Blüthgen C, Milanese G et al (2021) Lung nodules in melanoma patients: morphologic criteria to differentiate non-metastatic and metastatic lesions. Diagnostics 11:837. 10.3390/diagnostics11050837 34066913 10.3390/diagnostics11050837 PMC8148527

[20] Barnett J, Pulzato I, Wilson R et al (2019) Perinodular vascularity distinguishes benign intrapulmonary lymph nodes from lung cancer on computed tomography. J Thorac Imaging 34:326–328. 10.1097/RTI.0000000000000394 30664064 10.1097/RTI.0000000000000394

[21] Fu BJ, Lv FJ, Li WJ, Lin RY, Zheng YN, Chu ZG (2021) Significance of intra-nodular vessel sign in differentiating benign and malignant pulmonary ground-glass nodules. Insights Imaging 12:65. 10.1186/s13244-021-01012-7 34037864 10.1186/s13244-021-01012-7 PMC8155149

[22] Caparica R, Mak MP, Rocha CH et al (2016) Pulmonary nodules in patients with nonpulmonary cancer: not always metastases. J Glob Oncol 2:138–144. 10.1200/JGO.2015.002089 28717693 10.1200/JGO.2015.002089 PMC5495454

[23] Khokhar S, Vickers A, Moore MS, Mironov S, Stover DE, Feinstein MB (2006) Significance of non-calcified pulmonary nodules in patients with extrapulmonary cancers. Thorax 61:331–336. 10.1136/thx.2005.051508 16467070 10.1136/thx.2005.051508 PMC2104619

[24] Jin K, Wang K, Zhang H et al (2018) Solitary pulmonary lesion in patients with history of malignancy: primary lung cancer or metastatic cancer? Ann Surg Oncol 25:1237–1244. 10.1245/s10434-018-6360-6 29417404 10.1245/s10434-018-6360-6

[25] Parkar AP, Kandiah P (2016) Differential diagnosis of cavitary lung lesions. J Belg Soc Radiol 100:100. 10.5334/jbr-btr.1202 30151493 10.5334/jbr-btr.1202 PMC6100641

[26] Giacomelli IL, Barros M, Pacini GS et al (2019) Multiple cavitary lung lesions on CT: imaging findings to differentiate between malignant and benign etiologies. J Bras Pneumol 46:e20190024. 10.36416/1806-3756/e20190024 31859704 10.36416/1806-3756/e20190024 PMC7462708

[27] Chaudhuri MR (1973) Primary pulmonary cavitating carcinomas. Thorax 28:354–366. 10.1136/thx.28.3.354 4353362 10.1136/thx.28.3.354 PMC470041

[28] Gafoor K, Patel S, Girvin F et al (2018) Cavitary lung diseases: a clinical-radiologic algorithmic approach. Chest 153:1443–1465. 10.1016/j.chest.2018.02.026 29518379 10.1016/j.chest.2018.02.026

[29] Van Riel SJ, Sánchez CI, Bankier AA et al (2015) Observer variability for classification of pulmonary nodules on low-dose CT images and its effect on nodule management. Radiology 277:863–871. 10.1148/radiol.2015142700 26020438 10.1148/radiol.2015142700

[30] Yoon SH, Kim YJ, Doh K et al (2021) Interobserver variability in Lung CT Screening Reporting and Data System categorisation in subsolid nodule-enriched lung cancer screening CTs. Eur Radiol 31:7184–7191. 10.1007/s00330-021-07800-5 33733688 10.1007/s00330-021-07800-5

[31] Nair A, Bartlett EC, Walsh SLF et al (2018) Variable radiological lung nodule evaluation leads to divergent management recommendations. Eur Respir J 52:1801359. 10.1183/13993003.01359-2018 30409817 10.1183/13993003.01359-2018

[32] Zhong F, Liu Z, An W et al (2022) Radiomics study for discriminating second primary lung cancers from pulmonary metastases in pulmonary solid lesions. Front Oncol 11:801213. 10.3389/fonc.2021.801213 35047410 10.3389/fonc.2021.801213 PMC8761898

[33] Stereotactic ablative body radiation therapy (SABR): a resource. UK SABR Consortium guidelines v6.1 Jan 2019. Available via www.sabr.org.uk

[34] Shelton J, Zotow E, Smith L et al (2024) 25-year trends in cancer incidence and mortality among adults aged 35–69 years in the UK, 1993–2018: retrospective secondary analysis. BMJ 384:e076962. 10.1136/bmj-2023-076962 38479774 10.1136/bmj-2023-076962 PMC10935512

[35] Baldwin DR, Gustafson J, Pickup L et al (2020) External validation of a convolutional neural network artificial intelligence tool to predict malignancy in pulmonary nodules. Thorax 75:306–312 32139611 10.1136/thoraxjnl-2019-214104 PMC7231457

[36] O’Dowd E, Berovic M, Callister M et al (2024) Determining the impact of an artificial intelligence tool on the management of pulmonary nodules detected incidentally on CT (DOLCE) study protocol: a prospective, non-interventional multicentre UK study. BMJ Open 14:e077747. 10.1136/bmjopen-2023-077747 38176863 10.1136/bmjopen-2023-077747 PMC10773382

