# Supplementary material for: Semantic CT features and differentiation model: new primary lung cancer versus metastasis after previous malignancy
Source: Eur Radiol. 2026 May 6;36(9):6969–80. doi: 10.1007/s00330-026-12557-w (PMC13451270; doi:10.1007/s00330-026-12557-w)
Supplement: Supplementary file 1 [file 330_2026_12557_MOESM1_ESM.pdf]

Supplemental Figure Legends

Figure S1	AI-SONAR study recruitment criteria (IRAS 331656, ClinicalTrials.gov ID NCT05375591).
Figure S2	Definition of nodule/lesion ground truth in AI-SONAR study (IRAS 331656, ClinicalTrials.gov ID NCT05375591).
Figure S3	Baseline radiology concordance across all nine radiologists. Cohen's Kappa analysis comparing reader vs reader for each semantic feature. Table outlining Cohen's Kappa metrics and corresponding interpretation of agreement level between 2 readers (N =30).
Figure S4	Definition of central versus peripheral nodule/lesion topography using 'proximal bronchial tree' threshold as outlined in UK SABR Consortium 2019 guidelines[34].
Figure S5	Radar plot demonstrating semantic features present in percentage of cases (N=649) in both outcome classes (SPLC vs LM). Each ring = 25% quartile. Outwards deflection towards semantic feature indicates "Present or Yes". Abbreviations: LM:Lung Metastasis, SPLC:Second Primary Lung Cancer.
Figure S6	Histogram plot demonstrating distribution of lesion size (mm) in each outcome class. Abbreviations: LM:Lung Metastasis, SPLC:Second Primary Lung Cancer.

Inclusion Criteria:

- Age>18 years
- Confirmed history of prior radically treated or curative-intent treated solid organ cancer <10 years of new index CT thoracic imaging demonstrating a new pulmonary nodule/lesion with either of the following
    - Biopsy confirming previous malignancy with MDT consensus and successful cancer resolution/remission following anti-cancer treatment on interval imaging, blood assay analysis or clinically with baseline pre-treatment CT Thorax showing no evidence of metastatic lung disease
    - Where biopsy was not possible/confirmed for previous malignancy, MDT consensus outcome confirming cancer (+/- calculated Herder score >80% if applicable) and decision to treat as malignancy with subsequent resolution/remission following anti-cancer treatment on interval imaging or blood assay analysis or clinically with baseline pre-treatment CT Thorax showing no evidence of metastatic lung disease

- Radical/curative intent treatment defined as any of the following:
    - Surgical resection
    - Radical radiotherapy or stereotactic beam radiotherapy
    - Radical chemotherapy
    - Radical chemo-radiotherapy
    - Any new novel therapy approach +/- combination in line with UK national guidelines and defined as 'radical/curative intent
    - Multi-modality treatment with any of the above

- New pulmonary nodule/lesion ground truth known
    - Scan data showing size stability for 2 years (diameter or volumetry) or resolution
    - Scan data showing progressive nodule/lesion enlargement or increase in number on interval imaging with MDT consensus +/- PET with Herder score >80% (if applicable) determining malignant disease
    - Biopsy sampling confirming nodule/lesion aetiology

- CT scan slice thickness ≤2.5mm
- Nodule/lesion ≥5mm in diameter

Exclusion Criteria:

- CT imaging>10 years old
- Non-solid haematological malignancy
- Cases of radically treated primary cancer disease with early oligometastatic recurrence treated radically

Figure S1. AI-SONAR study recruitment criteria (IRAS 331656, ClinicalTrials.gov ID NCT05375591).

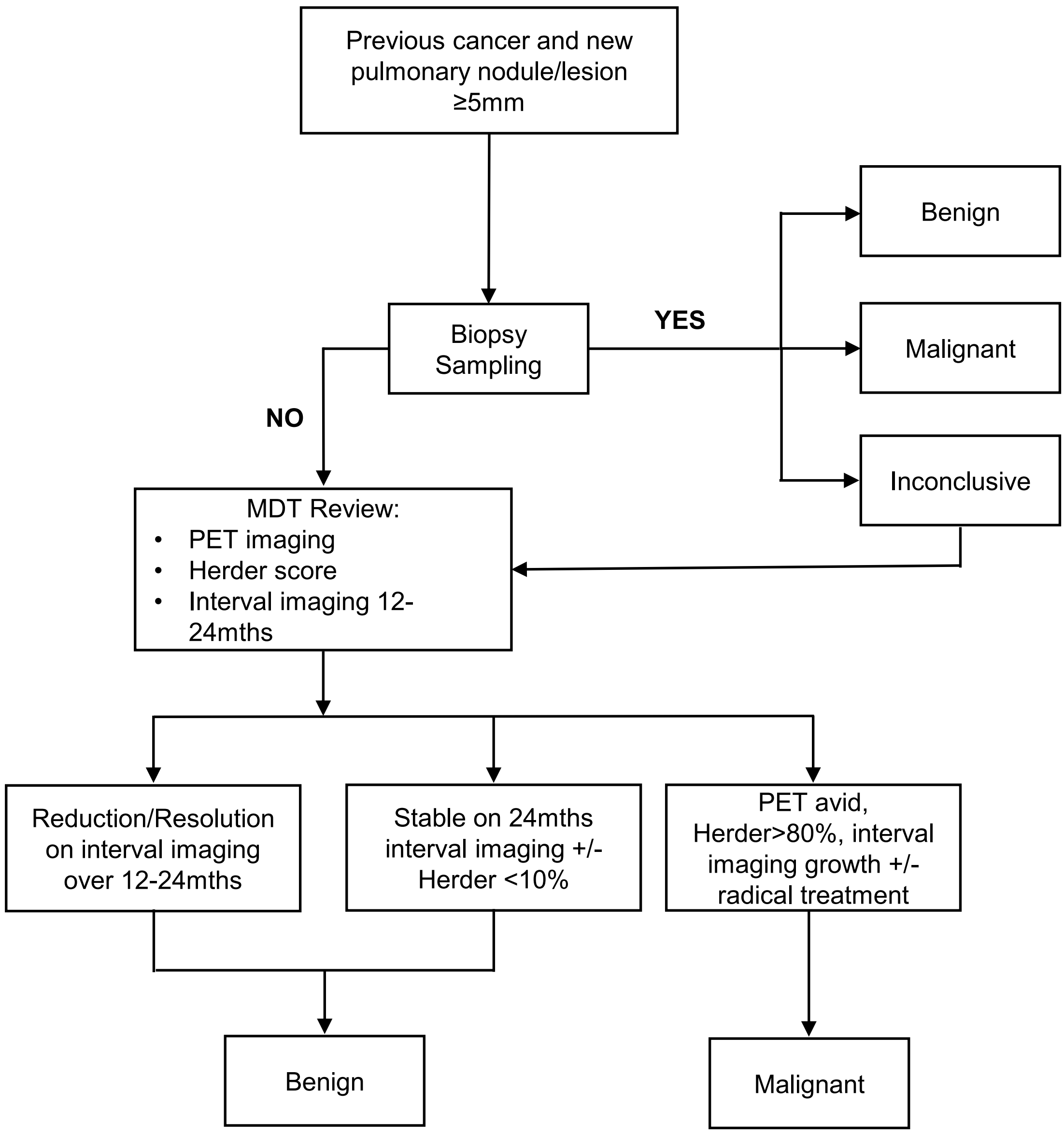

Figure S2. Definition of nodule/lesion ground truth in AI-SONAR study (IRAS 331656, ClinicalTrials.gov ID NCT05375591).

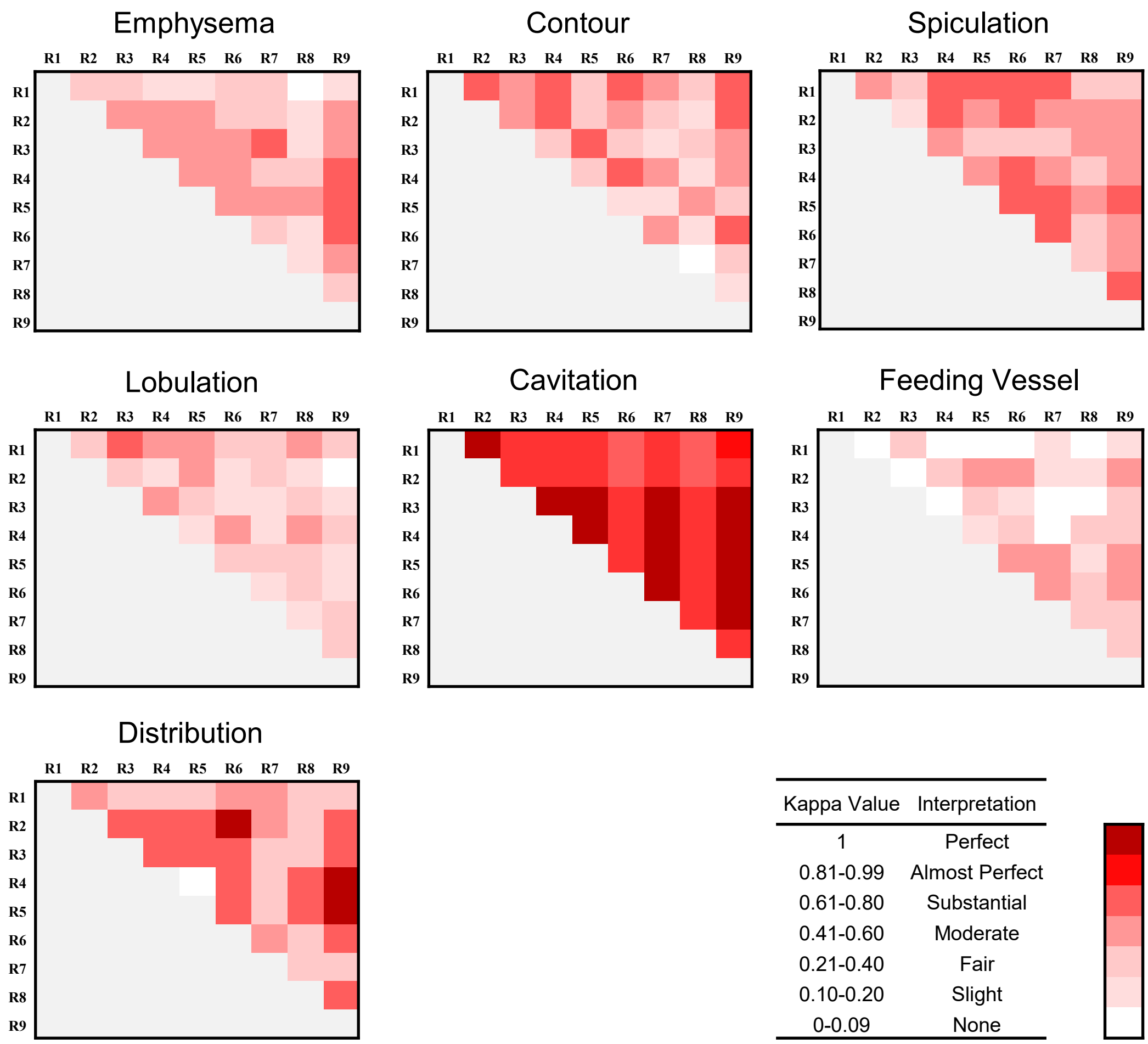

Kappa Value	Interpretation
1	Perfect
0.81-0.99	Almost Perfect
0.61-0.80	Substantial
0.41-0.60	Moderate
0.21-0.40	Fair
0.10-0.20	Slight
0-0.09	None

Figure S3. Baseline radiology concordance across all nine radiologists. Cohen's Kappa analysis comparing reader vs reader for each semantic feature. Table outlining Cohen's Kappa metrics and corresponding interpretation of agreement level between 2 readers (N =30).

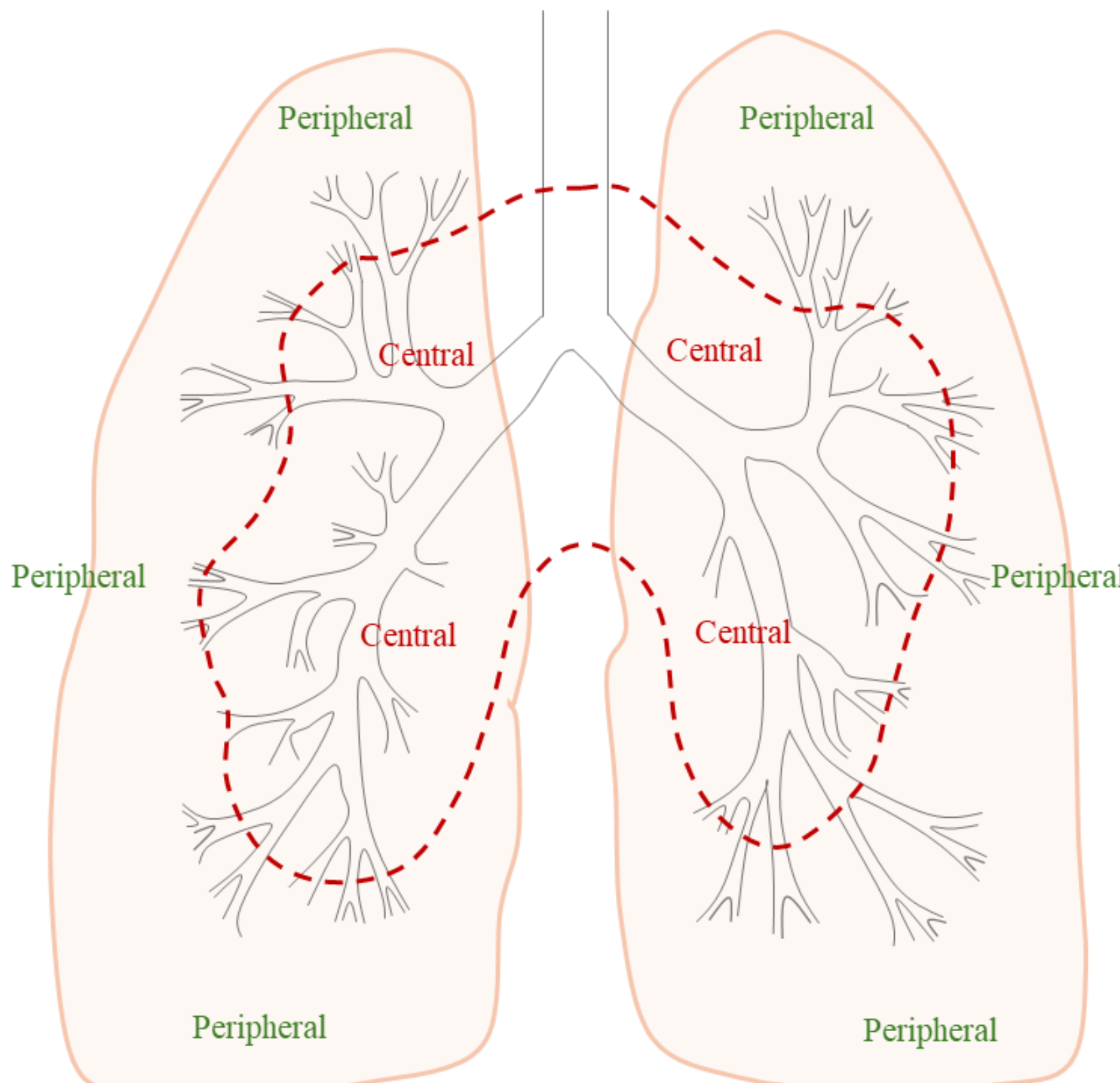

Figure S4. Definition of central versus peripheral nodule/lesion topography using 'proximal bronchial tree' threshold as outlined in UK SABR Consortium 2019 guidelines[34].

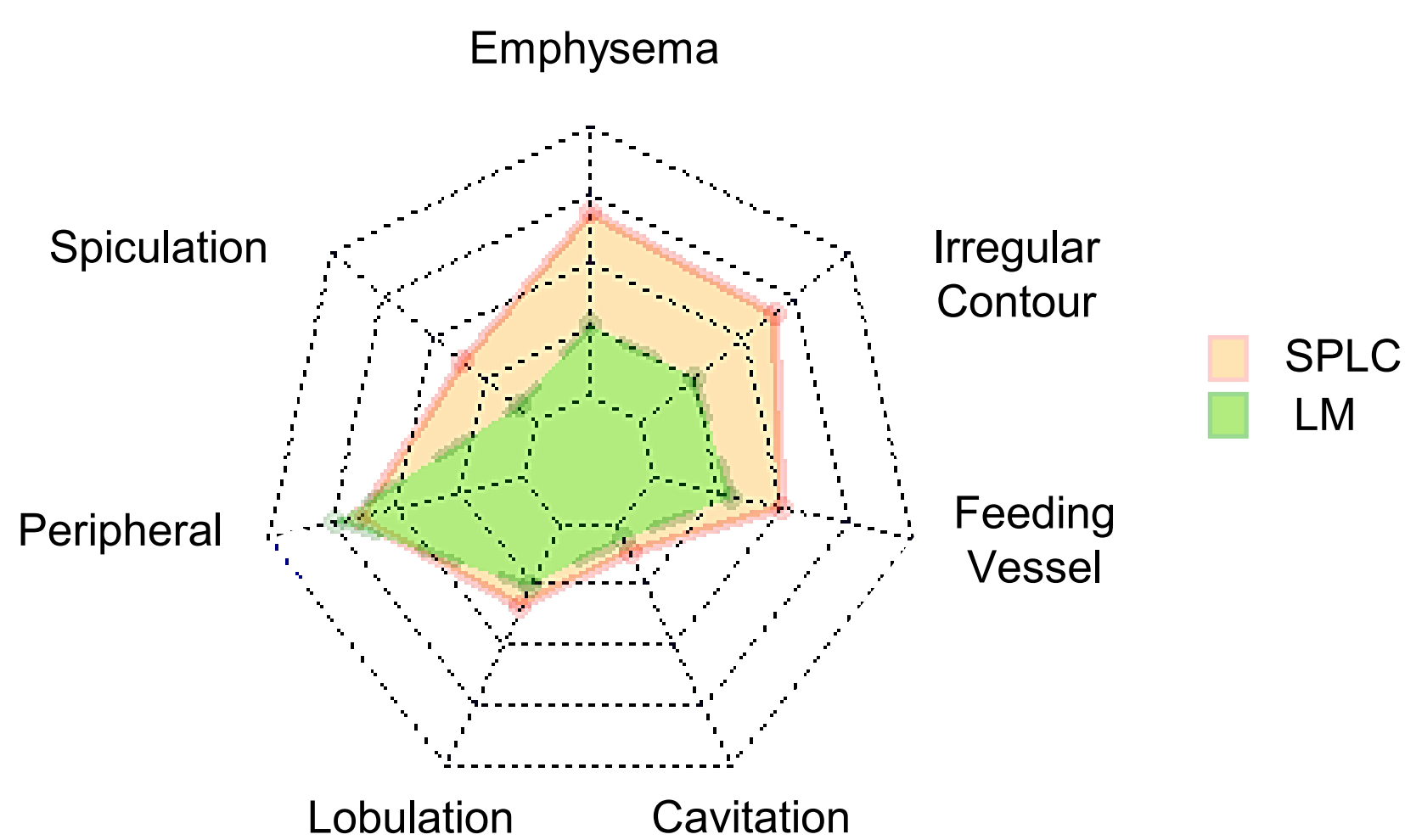

Figure S5. Radar plot demonstrating semantic features present in percentage of cases (N=649) in both outcome classes (SPLC vs LM). Each ring = 25% quartile. Outwards deflection towards semantic feature indicates “Present or Yes”. Abbreviations: LM:Lung Metastasis, SPLC:Second Primary Lung Cancer.

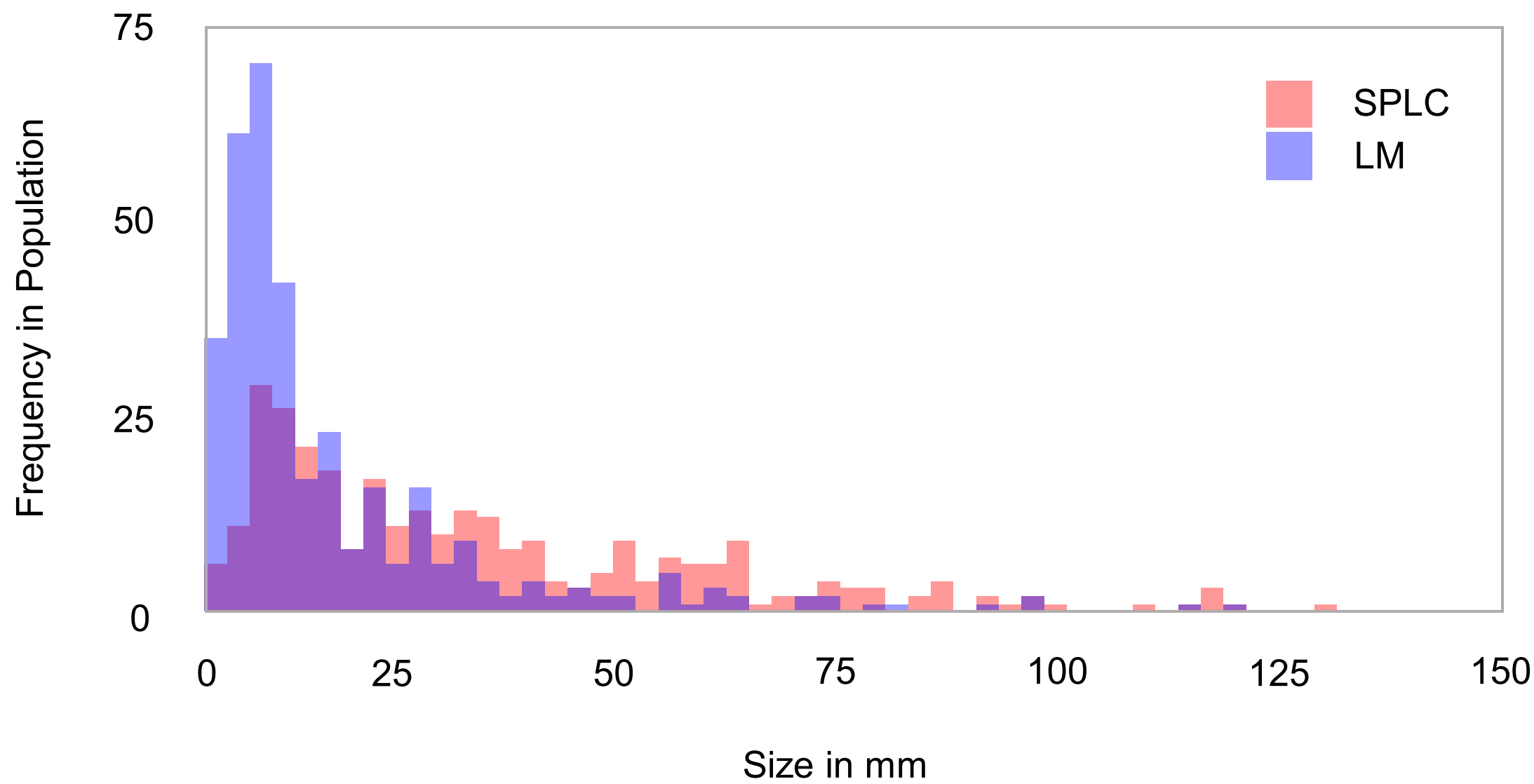

Figure S6. Histogram plot demonstrating distribution of lesion size (mm) in each outcome class. Abbreviations: SPLC:Second Primary Lung Cancer, LM:Lung Metastasis.
